# Effects of Mind-Body Interventions on Adolescents’ Cooperativeness and Emotional Symptoms

**DOI:** 10.3390/bs12020033

**Published:** 2022-02-02

**Authors:** Susanna Feruglio, Stefania Pascut, Alessio Matiz, Andrea Paschetto, Cristiano Crescentini

**Affiliations:** 1 Department of Languages and Literatures, Communication, Education and Society, University of Udine, 33100 Udine, Italy; stefania.pascut@uniroma1.it (S.P.); alessio.matiz@uniud.it (A.M.); andrea.paschetto@uniud.it (A.P.); cristiano.crescentini@uniud.it (C.C.); 2 Department of Psychology, Sapienza University of Rome, 00118 Rome, Italy; 3 WHO Healthy City Project—Municipality of Udine, 33100 Udine, Italy; 4 Institute of Mechanical Intelligence, Scuola Superiore Sant’Anna di Pisa, 56010 Pisa, Italy

**Keywords:** adolescence, mindfulness meditation, autogenic training, character, cooperativeness, emotional symptoms

## Abstract

Background: Mind-body interventions may support the development of adolescents’ self-regulation and provide a protective effect against maladaptive outcomes, e.g., internalizing and externalizing problems. The present study aimed at evaluating the effects of mindfulness-oriented meditation training (MOM) and autogenic training (AT) on a group of healthy Italian adolescents’ character dimensions, emotional and behavioral difficulties. Methods: 72 adolescents were randomly assigned to MOM/AT conditions and tested before and after the 8-week trainings through self-report measures (Temperament and Character Inventory 125, TCI; Strengths and Difficulties Questionnaire for Adolescents, SDQ-A). Main analyses involved robust and repeated measures ANOVAs, carried out separately for character TCI and SDQ-A scales. Results: After trainings, we found increased levels of cooperativeness and reduced emotional symptoms. Changes in these dimensions were negatively correlated: the more participants increased in their cooperativeness the greater decrease they showed in emotional symptoms. Conclusion: Both MOM and AT enhanced a cooperative attitude in adolescents and helped reducing their emotional problems. Therefore, it may be useful to apply these mind-body interventions in school settings as they can have a protective effect on the well-being and psychosocial adjustment of youths, through fostering their character maturity and helping them to better regulate their emotions.

## 1. Introduction

Adolescence is a period of development and change for youths across multiple domains [1]. Exposure to some stressors increases as youths age into adolescence [2] and this has been associated with maladaptive outcomes, including internalizing problems, externalizing behaviors, academic difficulties, and health risk behaviors [3,4]. Increasingly, there are calls for further investigation on how to reduce the maladaptive aspects of stress experienced in adolescence which are often related to long-term problems, as well as the risk of developing psychological difficulties in adult life; for example, anxiety, depression and eating disorders typically emerge in mid-late adolescence and tend to become chronic [5,6]. The impact of such determinants can be mitigated by protective factors, such as good adaptive coping and emotion regulation strategies, prosocial behavior and social connectedness, as well as support from schools and mental health services [7,8,9].

School-based prevention programs offer a means of targeting a broad portion of the population at or before the peak emergence of critical conditions, especially with programs offered to all students, thus avoiding the disadvantages of selecting out at-risk individuals, e.g., lack of failsafe screening and potential stigmatization [10,11]. Interventions that reinforce adolescents’ capacity for coping with stressful circumstances, by targeting the emotional and attentional processes associated with stress, may support the development of adolescents’ self-regulation and provide a protective effect against maladaptive outcomes, including internalizing problems and externalizing behaviors [12,13]. Self-regulation (SR) is a psychological construct which encompasses a range of important competencies, including the capacity for controlling one’s emotions, the ability to have positive interactions with others, and the capacity for avoiding inappropriate or aggressive actions [14]. Particularly, SR of negative emotions [15], such as anger and sadness, is related to social and peer acceptance across childhood and adolescence. Emotion regulation is regarded as a fundamental aspect of many kinds of youth psychopathology [16] and a potential mediator of the relationship between exposure to risk and healthy developmental outcomes for minority youth [17]. There is growing evidence that SR and emotion regulation play an important foundational role in development and maintenance of physical health and well-being in childhood and across the lifespan [18,19]. A systematic review and meta-analysis of 49 randomized clinical trials evaluating SR interventions (e.g., curriculum-based, yoga and mindfulness, social and personal skills, and exercise-based interventions), found that these interventions were effective in children and adolescents (0–19 years), contributing to positive outcomes on health and social measures, such as academic achievement, social skills, mental health, reducing behavioral problems, conduct disorders, school suspensions and substance abuse [20].

In particular, mindfulness-based programs have been broadly theorized to improve SR of emotions, behavior, and cognitive processes. Mindfulness has been defined as “paying attention in a particular way: on purpose, in the present moment, and non-judgmentally” [21]. The use of mindfulness meditation practices to reduce suffering and distress has been a feature of many Eastern philosophical traditions (e.g., Buddhism) for hundreds of years, and has been increasingly used in Western medicine for well over 30 years [22,23]. Meditation practices emerged from a body of Buddhist and other contemplative traditions and represent a complementary approach to mental health and well-being that combines psychological approaches to reduce stress and discomfort, including cognitive–behavioral therapies [24,25,26], and relaxation techniques. Most of the research regarding mindfulness approaches has been conducted with adults and in professional or therapeutic contexts [27,28,29]. More recently, mindfulness-based interventions have been embraced and widely disseminated in schools [30,31,32,33], but there are still insufficient methodologically robust studies to make definitive conclusions about efficacy [34,35]. Along with mindfulness there is a wide variety of mind-body interventions that share some common goals and positive outcomes amongst young people, e.g., numerous studies have found that relaxation training correlates with reduced stress, improved symptom management and quality of life [36,37,38] and improvements in externalizing, emotional and behavioral symptoms [39,40]. Until now, few studies were designed to compare the effects of different mind-body approaches in adults or in adolescents and those studies showed contrasting results. For example, some studies [41,42] compared mindfulness meditation and different types of relaxation training (e.g., muscle relaxation, autogenic relaxation), with control groups. One study showed that both types of training had similar beneficial effects in reducing mental distress and improving a positive state of mind, while mindfulness meditation specifically reduced ruminative and distractive thoughts compared to relaxation practices [41]; in contrast, another study found no effects of mindfulness and somatic relaxation in the experience of stress and the use of coping strategies in medical students [42].

To further address this issue, the present study aimed to evaluate the effects of two mind-body interventions on adolescents, namely mindfulness-oriented meditation (MOM) and autogenic training (AT), a relaxation technique first introduced by the German psychiatrist Johannes Heinrich Schultz in 1932. AT consists of a series of relaxation exercises, in conjunction with verbal cues, to learn to regulate physical processes (e.g., breathing and warmth) to induce a state of relaxation. Both programs may positively impact adolescents’ psychosocial adjustment; more specifically, we predict that these types of training will show some beneficial effect in reducing emotional and behavioral problems in youth.

Moreover, as shown in previous studies, cognitive, emotional and social experiences could be affected by personality characteristics. In this regard, we built on previous research suggesting the importance of personality traits as protective factors for adolescents’ well-being and psychosocial functioning and the usefulness of assessing these dimensions, especially in the context of interventions that may potentially enhance some character dimensions [43]. According to the well-known temperament and character inventory of personality (TCI; [44,45]), the character, i.e., the maturity of the self, is defined at three levels: at the intrapersonal level by self-directedness (SD; the tendency to be purposeful, responsible and reliable vs. purposeless, blaming and unreliability), at the interpersonal level by cooperativeness (C; the attitude of being helpful, empathic and ethical vs. unhelpful, critical and opportunistic), and at the transpersonal level by self-transcendence (ST; the tendency to be holistic and united with the universe vs. self-centered and unimaginative). A restricted number of studies focusing on character strengths using TCI shows that adolescents who report high levels of persistence and character maturity (high scores in SD and C) [46], report low levels of depressive symptoms [46,47] and decreased risks of developing psychiatric disorders during early adulthood [48]. Previous studies also found that maturation of the self in aspects related to empathy, kindness, and forgiveness (C) was positively associated with pro-social behaviors and more marginally and negatively with total difficulties and externalizing problems [43]. Overall, these data indicate that another key element for understanding well-being and psychosocial adjustment in adolescence seems to involve the possibility to experience satisfying interactions with other individuals [49]. Thus, we took these dispositional factors into consideration; we measured changes in character dimensions and the psychosocial adjustment of adolescents before and after their attendance on a mindfulness-based and an AT course. We predicted finding some positive changes in these measures after the 8-week trainings, as a result of enhanced self-regulatory processes in youth.

## 2. Materials and Methods

### 2.1. Procedure

The research study involved seventy-two adolescents aged between 16 and 20 years recruited in two five-year high schools, located in two small cities of approximately 7000 and 14,000 inhabitants in the north-east of Italy (i.e., Lignano Sabbiadoro and Latisana). Students belonged to four classes: the 3rd and 4th grade of a technical high school, and the 3rd and 4th grade of a scientific and linguistic high school. The schools were randomly assigned to a MOM or an AT condition, resulting in one 3rd and one 4th grade class assigned to each training. The training programs were led by two expert instructors of mindfulness meditation and autogenic practices, who were both present in each group meeting (more specifically, one expert conducted the MOM practices and the other conducted the AT practices; they held all group discussions, which took place after the practices, together). The participants were tested twice, before the beginning and after the end of the courses (Session 1: first weeks of March 2017, and Session 2: last weeks of May 2017), through two self-report measures, the TCI and SDQ-A. Questionnaires were administered in groups in participants’ classrooms, in the presence of their teacher and a research psychologist. The courses were designed to be comparable and structurally equivalent; they lasted 8 weeks, during which participants attended a group session once per week. Group sessions for both training programs took place during morning school time, on consecutive days. All the subjects were invited to continue to practice daily at home and to record in a diary each individual session. This study was approved by the Ethics Committee of the University of Udine (Ethical Application Ref: CGPER-2016-11-23-01) and all procedures performed in the study were in accordance with the ethical standards of the 1964 Helsinki declaration and its later amendments.

### 2.2. Participants

Seventy-two adolescents aged between 16 and 20 years (M = 17.5 ± 0.75; 63.9% females) participated in the study. In total, 69 participants completed the training and the research questionnaires. We thus excluded 3 participants who had not concluded the training or the research questionnaires. All participants were volunteers and were previously informed about the research purposes according to procedures reviewed by their schools. More specifically, the study methodology, aims and procedures were presented to teachers, parents and students in a school general assembly by the experts, with the opportunity to ask questions and seek clarification from the researchers. All recruited participants reported no past history of neurological or mental illness and had no previous experience with MOM and AT training or with the outcome measures used in the study.

### 2.3. Trainings

The mindfulness-oriented meditation training (MOM) [50,51,52] was an 8-week course in which participants attended a 2 h group session once per week. In each group-session they were taught some theoretical aspects related to mindfulness (e.g., attention regulation, equanimity, defusion, etc.), then they practiced 30 min of meditation (10 min focus on breath, 10 min body scan and 10 min observing contents of the mind), and finally they had a group discussion about meditation practice. The participants were also asked to meditate autonomously at home every day, following an audio-recorded meditation equivalent to that performed during group meetings, and to record each session in a diary. The general purpose of mindfulness-based meditation is to cultivate a state of open, non-judgmental, and not necessarily relaxing, awareness in the present moment.

The autogenic training (AT) [53] was structurally equivalent to the MOM course. In the 2 h group-sessions, participants were invited to use physical processes, in conjunction with verbal cues, to relax and learn to regulate some autonomic activities and internal processes, including breathing and warmth, paying attention to the heartbeat, abdominal sensations, and the coolness of the forehead. After a theoretical introduction lasting up to 30 min and 30 min of guided practice, participants had a group discussion. All the participants were asked to continue to practice daily the same exercises practiced in group and to record each session in a diary. The general purpose of autogenic practice is to enhance a state of calmness and relaxation.

### 2.4. Self-Report Measures

#### 2.4.1. Temperament and Character Inventory-125

Personality was assessed by the 125-item version of the self-report form of the TCI [45], administered using the Italian adaptation [54]. The TCI-125 operationalizes Cloninger’s personality model [44] with items organized into four temperament scales and three character scales. For the purpose of the present study, we report here the results concerning the character dimensions, namely SD, implying an autonomous self-concept and feelings of hope, honor and self-confidence; C, involving interindividual differences in the acceptance of others, compassion, and charity; and ST, consisting of creative self-forgetfulness, transpersonal identification, and spiritual acceptance. Character traits refer to interindividual differences in the conceptual representation of the self in relation to other people and to the external world and may be related to the functioning of higher cognitive systems [45,55]. Each item requires a True or False response and a higher final score for each scale corresponds to higher levels of SD, CO and ST traits. The three TCI character scales showed good psychometric properties in Italian adolescents: SD α = 0.75, C α = 0.81, ST α = 0.72 [54]. In addition, in our sample the average Cronbach’s alpha of Session 1 and Session 2 was good for SD (α = 0.85), CO (α = 0.82), and ST (α = 0.77).

#### 2.4.2. Strengths and Difficulties Questionnaire—Adolescents Version

The self-report form SDQ-A [56,57] was used as a reliable measure covering the most important domains of psychopathology in a normal school-age population. The rationale for the scales was based on nosological criteria from the Diagnostic and Statistical Manual of Mental Disorders (4th ed., DSM-IV) [58]. The SDQ-A is composed of 25 items, organized in five 5-item scales: Emotional symptoms, (EMO, example item: ‘I am often unhappy, depressed or tearful’), Peer problems (PEE, example item: ‘I am usually on my own’), Conduct problems (BEH, example item: ‘I am often accused of lying or cheating’), Hyperactivity/Inattention (HYP, example item: ‘I am restless. I cannot stay still for long’), and Prosocial behaviors (PRO, example item: ‘I am helpful if someone is hurt, upset or feeling ill’). All items are rated on a 3-point rating scale (0 for “not true,” 1 for “somewhat true” and 2 for “certainly true”). The total scores of each scale range from 0 to 10. Higher scores correspond to higher levels of difficulties in EMO, BEH, HYP, PEE scales and to a higher propensity for prosocial behaviors for the PRO scale. The SDQ-A scales showed acceptable psychometric properties in adolescents for EMO (α = 0.66), BEH (α = 0.60), HYP (α = 0.67) and PRO (α = 0.66), while PEE showed poor internal consistency reliability (α = 0.41) [57]. In our sample the mean Cronbach’s alpha of Session 1 and Session 2 was good for EMO (α = 0.77), it was acceptable for HYP (α = 0.62) and PRO (α = 0.64), while it was poor for BEH (α = 0.59) and PEE (α = 0.53).

#### 2.4.3. Daily Practice Diaries

All participants attending the MOM and AT programs were asked to complete diaries on daily practice. More specifically, they were asked to report daily if they practiced individually at home, for how long and at what time of the day. They were also free to report any comments or personal experiences about their practice that they might want to share during the following group discussion.

### 2.5. Data Analysis

Continuous measures were summarized reporting mean and standard deviation of raw data obtained before and after trainings in the MOM and AT groups (Table 1). One participant of the AT group did not fill in the TCI character scales at Session 1 and one participant of the MOM group did not fill in the PEE scale at Session 2, thus these two subjects were excluded from the relative analyses (TCI character and PEE scales). For all other participants, the response rate was 99.7% for TCI and 99.8% for SDQ-A; scale-mean substitution was used to manage missing items. We compared the rates of group meeting attendance and the mean practice time (reported in minutes) in the MOM and AT groups using two t-tests for independent samples. Then, we tested the distribution of each questionnaire scores for normality with the Shapiro–Wilk’s W test. The main analysis concerned a series of repeated measures 2 × 2 ANOVAs and 2 × 2 ANOVAs using robust M-estimators with 2000 bootstraps for not normally distributed data, carried out separately for the different TCI character scales (SD, C, ST) and the SDQ-A scales (EMO, BEH, HYP, PEE, PRO), with Session (Session 1 and Session 2) as a within-subjects factor and Group (MOM and AT) as a between-subjects factor. Correlational analysis was employed to examine the relationship between post-pre training changes of the TCI and SDQ-A measures where significant differences were observed in the before vs. after training comparison of means. Overall, the conventional level of *p* < 0.05 was used as a statistical threshold of significance. All analyses were conducted using JASP (version 0.16) and R (version 3.6.3) software.

## 3. Results

### 3.1. Training Attendance and Practice Time

Before performing the main analyses, we compared the two groups in terms of attendance at group meetings and individual practice time: participants to the MOM group had, on average, 1.19 absences (sd = 0.90), while the mean for the AT group was 0.59 (sd = 0.93); the total individual practice time accumulated during the training was, on average, 500.69 min (sd = 217.20) for the MOM group, and 600.57 min (sd = 434.36) for the AT group. The MOM group had a significantly higher absence rate (*t* = −2.691, df = 67, *p* = 0.009), but the two groups did not differ significantly in total practice time reported by participants (*t* = 1.179, df = 67, *p* = 0.24).

### 3.2. Changes in Character Traits 

**Figure 1:**
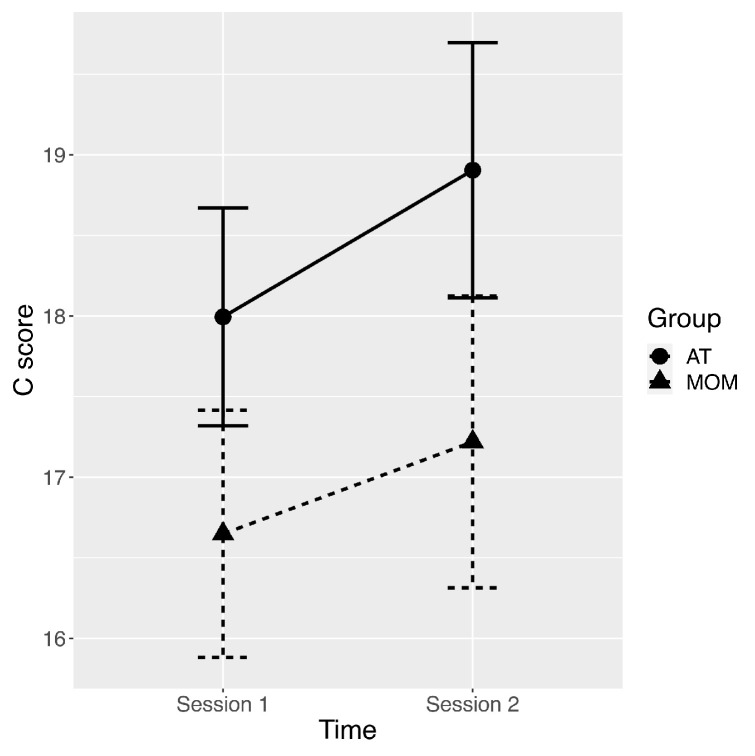
Plot of average scores in Cooperativeness with error bars representing standard errors of the mean for pre-training assessment (Session 1) and post-training assessment (Session 2), and for AT (autogenic training) and MOM (mindfulness-oriented meditation) groups.

A Shapiro–Wilk’s test showed that the TCI character scores of C and ST scales were not normally distributed at Session 1 in the AT group (*p* < 0.05). On the basis of two 2 (Session: Session 1 and Session 2) × 2 (Group: MOM and AT) ANOVAs using robust M-estimator and 2000 bootstraps carried out on C and ST scores, there was a significant main effect of Session for the C scale (Session 2 > Session 1; ψ = −1.1, *p* = 0.001, see Table 1 and Figure 1). On the basis of a 2 (Session: Session 1 and Session 2) × 2 (Group: MOM and AT) repeated measures ANOVA carried out on SD scores, there was a significant main effect of Group (AT > MOM; F [1,66] = 4.861, *p* = 0.031; ηp^2^ = 0.069, see Table 1). No other main effects or interactions were significant (all *p* > 0.1). Thus, MOM and AT trainings led to higher scores on the cooperativeness scale at Session 2 than at Session 1.

### 3.3. Changes in Strengths and Difficulties Questionnaire

A Shapiro–Wilk’s test showed that at Session 1, the SDQ-A scores of the BEH, HYP, PRO scales in the MOM group, as well as the PEE scores in the AT group, were not normally distributed (all *p* < 0.05). Furthermore, at Session 2, the SDQ-A scores of the EMO scale were not normally distributed for the AT group (*p* < 0.05).

**Figure 2:**
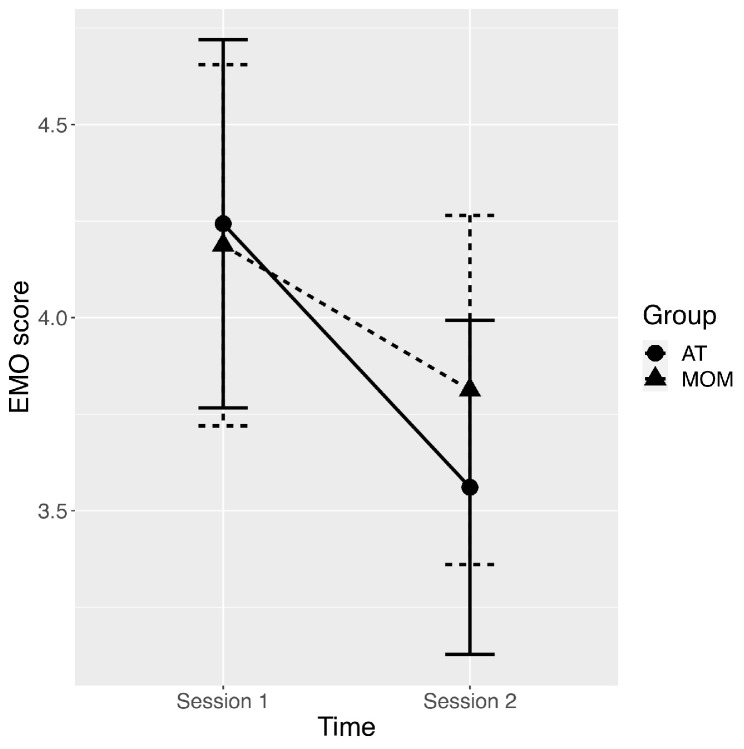
Plot of average scores in Emotional symptoms with error bars representing standard errors of the mean for pre-training assessment (Session 1) and post-training assessment (Session 2), and for AT (autogenic training) and MOM (mindfulness-oriented meditation) groups.

On the basis of five 2 (Session: Session 1 and Session 2) × 2 (Group: MOM and AT) ANOVAs using robust M-estimators and 2000 bootstraps carried out on each SDQ-A scale score (EMO, BEH, HYP, PRE, PRO), there was a significant main effect of Session for the EMO scale (Session 1 > Session 2; ψ = 0.8, *p* = 0.002, see Table 1 and Figure 2). No other main effects or interactions reached the significance threshold (all *p* > 0.06). Thus, after MOM and AT trainings, participants showed lower scores on the emotional symptoms scale.

### 3.4. Correlations

With regard to correlations between the indexes of Session 2 minus Session 1 changes in cooperativeness (C) and emotional symptoms (EMO), we obtained a significant negative correlation between changes in C and changes in EMO (Rho = −0.319, *p* = 0.008). The more participants increased in cooperativeness after the training the greater decrease they showed in emotional symptoms.

## 4. Discussion

The aim of this study was to investigate the effect of two 8-week mind-body interventions, namely MOM and AT, on adolescents’ character dimensions, emotional and behavioral difficulties. This was done by asking a sample of healthy late adolescents—recruited in two Italian five-year high-schools—to self-report their character traits and psychosocial adjustment (TCI and SDQ-A), before and after the trainings. Globally after both training programs, we found an increased level of cooperativeness, involving the acceptance of others, compassion and forgiveness. Alongside these changes, after the trainings, both groups showed decreased emotional symptoms, such as often feeling unhappy and downhearted. Moreover, the change in cooperativeness was negatively correlated with reduction in emotional problems: the more participants increased in their cooperativeness the more they showed a decrease in emotional symptoms after training.

Our study sample had already been investigated, at the “baseline” Session 1, in a previous study [43] that shed light on the relationship between temperament and character dimensions and different aspects of well-being and psychosocial functioning in adolescents. This study found that self-directness had a widespread protective effect on emotional and behavioral problems and that immaturity of the character (a combination of low self-directedness and low cooperativeness) showed a marked association with low psychological well-being and poor psychosocial adjustment. In line with the findings of the study by Crescentini et al. [43], we found that maturation of the self in aspects related to empathy and kindness was associated with enhanced psychological wellbeing in terms of a reduction in emotional problems. Overall, these data indicate the importance of considering personality traits, and character maturity in particular, as being closely connected to psychological adjustment and emotional regulation in youth.

Mindfulness and relaxation-based interventions based on autogenic training produced similar beneficial outcomes despite the different aims and techniques on which they focused, i.e., mindful awareness and relaxation practices, respectively. Similar findings emerged also in some previous studies [59,60]. The MOM and AT courses we used shared some important features: attendees of both training programs were encouraged to control their attention, to maintain a non-judgmental and non-reactive attitude, and to focus on breath and other physical sensations; moreover, the adolescents involved took an active role in the training through their daily individual practice and received support by sharing their challenges and successes with instructors and peers during the group meetings. It is possible that these common features of the two mind-body interventions were some of the key ingredients that promoted self-regulation, emotional well-being and changes in cooperativeness [61,62].

In general, the present results are consistent with what has emerged in previous research; implementing mind-body programs in schools can be an effective method to enhance positive intra- and interpersonal changes [63,64,65], such as self-regulation, coping, resilience [66,67,68] and emotional well-being [69,70], empathy, compassion and greater connection with others [71,72]. An emerging field of study—social and emotional learning (SEL)—highlights the importance of improving socio-emotional functioning and psychosocial adjustment in students, including recognition and management of emotions, empathy, and maintenance of positive interpersonal relationships, in order to support and maintain adolescents’ well-being [73,74,75] and to create a more pleasant and favorable school setting [74]. Thus, our research findings offer support to the field of SEL, showing that mind-body interventions, such as MOM and AT, can have a positive impact on socioemotional outcomes. Moreover, these interventions offer a means of targeting a broad section of the young population at or before the peak emergence of critical conditions, because they can, in theory, be offered to all students [10,11].

Our findings also show the importance of continuing to assess personality traits and their relation to well-being and psychosocial functioning in adolescence [72]. More specifically, it would be interesting for future studies to further investigate the effects of mind-body interventions on cooperative orientation. Recent research [76] involving five studies across different forms of cooperation, in both distributive and integrative negotiation contexts, and experimentally induced mindfulness, found substantial evidence that mindfulness is an effective intervention for increasing cooperation. Yet, these, and our, results are initial evidence that should be further investigated especially in youth. A larger body of evidence is available about the effects of mindfulness and relaxation on emotion regulation. Mindfulness has been shown to be effective in its regulatory function of targeting low positive emotionality, poor mood regulation and negative self-concept, which are risk factors implicated in the onset, development, and maintenance of depressive symptoms [77]. Several models, for example Shapiro’s [78] and Hölzel’s [79,80], outline both cognitive and experiential processes that enable emotional regulation through mindfulness meditation. Cognitive processes, for example, are involved in purposeful focusing of attention and reappraisal that allow for a new way of understanding and observing the transitory nature of emotional experience. With respect to AT, the existing literature is mainly focused on this kind of practice as a first intervention in therapeutic settings for children and adolescence. For example, some previous studies demonstrated how AT can be a valid self-help technique for adjustment disorders in young people, by diminishing the effects of, and easing the adaptation to, stress, and helping with recovery [81,82]. Other studies showed the effectiveness of AT in reducing anxiety and stress disorders [37,83]. A recent study [84] focused on how AT could help youth to cope with the pandemic; it reported strong beneficial effects of this practice on adolescents’ physical, psychological and relational health, recommending the practice of AT to people who experience anxiety, are afraid of illness, or feel that they have to improve the quality of their relationships with others.

Notwithstanding these promising results, more studies are needed to clearly define the specific effects and processes of the action of autogenic and mindfulness training in young people. Deepening this line of research will help tailor more effective programs for specific age groups and conditions, e.g., evaluating which delivery aspects of mind-body programs, such as duration of training, level of expertise of teacher training, environmental aspects, home practice, etc., induce the largest effects. Training should also be specifically adapted for use with youth, by shortening practice periods, making language appropriate for youth, and selecting age-appropriate activities, but further research is required to identify the optimal age, content and length of programs. These further investigations could be helpful to increase our knowledge on how to implement mind-body interventions in school settings with the aim of improving or preserving adolescents’ psychosocial functioning and well-being, which is becoming more and more important considering the challenges our society is facing and will face in the future.

The present findings should be interpreted in the context of some limitations. First, the exclusive use of self-report measures can be susceptible to socially desirable responding and these are sometimes considered less reliable than more objective measures, such as observation, in detecting, for instance, externalizing behaviors [85,86]. Other studies have showed that behavioral problems can reliably be assessed using self-reports [87], but it is advisable in the future to use multiple informants (e.g., adolescents, parents and teachers) to assess externalizing behaviors and to include implicit measures of personality which are more difficult to control or fake (e.g., the Implicit Association Test [50]). Moreover, we relied on daily diaries for the estimation of participants’ home practice, which is a common method used in mindfulness intervention studies [88], despite leaving unclear the extent to which this kind of measure relates to participants’ actual individual practice. Another limitation is that our results refer to a sample of adolescents living in the north-east of Italy and may not be generalizable to the larger population. Additionally, we lacked a passive, or waiting-list control group, which could have been useful to collect more informative data on the specific effects of the MOM and AT training. More studies are thus needed that include active and structurally equivalent control conditions to test training effects on specific mental health outcomes and behavioral change mechanisms in adolescents.

## 5. Conclusions

The aim of the present research was to investigate the impact of different mind-body interventions, specifically MOM and AT, on a group of Italian adolescents, by detecting potential changes in character dimensions, emotional and behavioral difficulties. Both training programs were effective in increasing participants’ level of cooperativeness, and in decreasing their emotional symptoms. These findings support the importance of implementing mind-body programs in schools as a valid method to enhance positive intra- and interpersonal changes in students. To conclude, it is suggested that consideration of the outcomes of this research could inspire future studies to further investigate the effectiveness and the underlying mechanisms of mindfulness and relaxation practices in young people, with the aim of promoting their healthy and harmonious growth.

## Figures and Tables

**Table 1:** Descriptive statistics of raw data obtained before and after trainings in the MOM and AT groups and results (main effects and two-way interaction) from ANOVA analyses regarding TCI character and SDQ-A scales.

		MOM Group (N = 32)		AT Group (N = 37)		2 × 2 Anova		
		Session 1 Mean SD	Session 2 Mean SD	Session 1 Mean SD	Session 2 Mean SD	Group ψ/F ^†^ *p*	Session ψ/F ^†^ *p*	Group: Session ψ/F ^†^ *p*
**TCI (Character)**	SD	13.50 5.45	12.81 5.91	15.43 5.51	16.42 5.50	4.86 ^†^ 0.031 *	0.08 ^†^ 0.77	2.62 ^†^ 0.11
	C	16.65 4.33	17.22 5.12	17.99 4.05	18.90 4.75	1.9 0.20	−1.1 0.001 *	−0.1 0.90
	ST	6.97 3.55	6.75 3.33	6.36 3.23	6.45 3.39	−0.4 0.66	0.0 0.81	−0.5 0.19
**SDQ-A**	EMO	4.19 2.64	3.81 2.56	4.24 2.90	3.56 2.63	0.3 0.71	0.8 0.002 *	0.7 0.12
	BEH	2.87 2.07	2.75 1.74	2.70 1.91	2.24 1.92	−0.6 0.16	0.1 0.48	0.4 0.24
	HYP	4.56 1.56	4.59 2.17	3.95 2.01	3.73 1.91	−0.6 0.17	−0.1 0.59	0.1 0.80
	PEE	2.97 2.10	3.29 2.30	2.78 2.00	2.62 1.30	−0.7 0.16	−0.2 0.44	0.0 0.96
	PRO	7.59 1.96	8.00 1.87	6.95 2.08	7.32 2.08	−1.0 0.06	−0.2 0.24	0.1 0.67

Notes: MOM = Mindfulness Oriented Meditation; AT = Autogenic Training; TCI = Temperament and Character Inventory; SD = Self-Directedness; C = Cooperativeness; ST = Self-Transcendence; SDQ-A = Strengths and Difficulties Questionnaire—Adolescent version; EMO = Emotional Symptoms; BEH = Conduct Problems; HYP = Hyperactivity/Inattention; PEE = Peer Problems; PRO = Prosocial Behaviors; * indicates statistically significant results (*p* < 0.05); ^†^ indicates F value.

## Data Availability

Data supporting the results are available from the corresponding author [SF], upon request.
